# Predicted 2100 climate scenarios affects growth and skeletal development of tambaqui (*Colossoma macropomum*) larvae

**DOI:** 10.1002/ece3.4429

**Published:** 2018-10-03

**Authors:** Ivã Guidini Lopes, Thyssia Bomfim Araújo‐Dairiki, Juliana Tomomi Kojima, Adalberto Luis Val, Maria Célia Portella

**Affiliations:** ^1^ Universidade Estadual Paulista (UNESP Univ Estadual Paulista) – Centro de Aquicultura da UNESP Jaboticabal Brazil; ^2^ Instituto Federal de Educação Ciência e Tecnologia do Amazonas Manacapuru Brazil; ^3^ Faculdade de Ciências Agrárias e Veterinárias (UNESP Univ Estadual Paulista) Jaboticabal Brazil; ^4^ Laboratório de Ecofisiologia e Evolução Molecular Instituto Nacional de Pesquisas da Amazônia (INPA) Manaus Brazil

**Keywords:** Amazon, carbon dioxide, fish larvae, IPCC, skeletal anomalies, temperature

## Abstract

Climate changes driven by greenhouse gas emissions have been occurring in an accelerated degree, affecting environmental dynamics and living beings. Among all affected biomes, the Amazon is particularly subjected to adverse impacts, such as temperature rises and water acidification. This study aimed to evaluate the impacts of predicted climate change on initial growth and development of an important Amazonian food fish, the tambaqui. We analyzed growth performance, and monitored the initial osteogenic process and the emergence of skeletal anomalies, when larvae were exposed to three climate change scenarios: mild (B1, increase of 1.8°C, 200 ppm of CO_2_); moderate (A1B, 2.8°C, 400 ppm of CO_2_); and drastic (A2, 3.4°C, 850 ppm of CO_2_), in addition to a control room that simulated the current climatic conditions of a pristine tropical forest. The exposure to climate change scenarios (B1, A1B, and A2) resulted in low survival, especially for the animals exposed to A2, (24.7 ± 1.0%). Zootechnical performance under the B1 and A1B scenarios was higher when compared to current and A2, except for condition factor, which was higher in current (2.64 ± 0.09) and A1B (2.41 ± 0.14) scenarios. However, skeletal analysis revealed higher incidences of abnormalities in larvae exposed to A1B (34.82%) and A2 (39.91%) scenarios when compared to current (15.38%). Furthermore, the bone‐staining process revealed that after 16 days posthatch (7.8 ± 0.01 mm total length), skeletal structures were still cartilaginous, showing no mineralization in all scenarios. We concluded that tambaqui larvae are well‐adapted to high temperatures and may survive mild climate change. However, facing more severe climate conditions, its initial development may be compromised, resulting in high mortality rates and increased incidence of skeletal anomalies, giving evidence that global climate change will hamper tambaqui larvae growth and skeletal ontogeny.

## 1 INTRODUCTION

The Amazon region is especially vulnerable to the effects of predicted climate change in the future, considering its great richness of species and characteristic seasonal environmental changes (IPCC, 2007, 2014). Increased temperatures and carbon dioxide (CO_2_) concentrations are constantly being registered all over the world and bring several concerns regarding the environment's dynamics and respective effects on plants and animals (Kochhann, Campos, & Val, 2015; Miller, Kroon, Metcalfe, & Munday, 2015; Oliveira & Val, 2017; Rosenzweig et al., 2008; Tseng et al., 2013). Likewise, reduced precipitation rates are expected in the Amazon region (IPCC, 2014), especially due to anthropogenic actions such as deforestation (Malhi et al., 2008), which directly affects this biome. The challenges that Amazonian biota will face with climate change may be overcome if the affected species display a well‐developed adaptive capacity, thus allowing them to survive in different environmental conditions (Hoffmann & Sgrò, 2011; Parmesan, 2006); otherwise, Amazonian biodiversity might be reduced.

As verified in several studies, both temperature and CO_2_ uptake in the oceans are increasing in a worrying scale, and by 2100, temperature may rise by up to 4°C, and pH is estimated to drop over 0.4 units (Caldeira & Wickett, 2003; IPCC, 2014). Thus, freshwater environments may also be affected, disturbing physiological mechanisms of living organisms, such as the ontogeny of organs and systems, growth and metabolism (Oliveira & Val, 2017; Pimentel et al., 2014; Rosa et al., 2012), leading to increased mortality rates (Ishimatsu, Kikkawa, Hayashi, Lee, & Kita, 2004). It is noteworthy that under stressful thermal conditions, aquatic organisms tend to be more susceptible to other stressors, such as water acidification (Byrne, 2011; Byrne, Soars, Selvakumaraswamy, Dworjany, & Davis, 2010; Rosa et al., 2013, 2014), especially younger organisms, due to the lack of a well‐developed physiological apparatus that aids the ionic balance. For instance, when body fluid's pH of fish decreases due to higher CO_2_ concentrations in the water, and the organism must compensate this change by accumulating HCO_3_^−^ ions (Ishimatsu et al., 2004).

The most challenging period of a fish's life cycle is the larval phase (Pimentel et al., 2014; Portella, Leitão, Takata, & Lopes, 2012). Ontogenetic processes are directly affected by the interaction between the developing fish and environmental conditions, such as water temperature and water acidification, which may hinder the development of different systems and body structures (Johnston & Hall, 2004; Pittman et al., 2013). During early life stages, fish are even more vulnerable to water acidification and temperature increases because the disruption of the acid‐base balance may lead to a compromised ontogeny of skeletal structures (Bignami, Sponaugle, & Cowen, 2013; Boglione et al., 2013; Georgakopoulou, Katharios, Divanach, & Koumoundouros, 2010; Ou et al., 2015; Pimentel et al., 2014; Pittman et al., 2013). Nevertheless, the appearance of skeletal malformations may have other possible causes, such as nutrition (Hamre et al., 2013) and genetic factors such as consanguinity (Izquierdo, Socorro, & Roo, 2010). In addition, if a genetic basis for a specific bone anomaly exists, this predisposition may be expressed when exposed to challenging environmental conditions (Kause, Ritola, & Paananen, 2007).

One of the most important fish species in the Amazon region is the tambaqui (*Colossoma macropomum*), which is, despite its ecological importance, highly explored in extractive fisheries and aquaculture, thus serving as an economic resource for several communities. As demonstrated in a previous study, future climate change will affect the physiological performance of tambaqui juveniles, possibly hindering its frequency in natural environments (Oliveira & Val, 2017). In this sense, tambaqui larvae may also be affected by such environmental changes, especially during its ontogeny, when its physiological and metabolic processes are not fully developed. We hypothesize that the exposure to different climate change scenarios with increased temperature and CO_2_ concentrations (IPCC, 2007) will disturb the ontogeny of tambaqui larvae, thus hampering its skeleton development. Therefore, the aim of this study was to investigate if these climatic changes would affect larvae growth, survival and skeleton ontogeny. The tambaqui belongs to the Ostariophysi group, characterized by the presence of cellular bones (which contains osteocytes) and the Weberian apparatus, an anatomic structure that bonds the swim bladder to the auditory system of these fish (Bird & Mabee, 2003; Estêvão et al., 2011). As the ossification processes that occur in this group are similar in several important species, such as zebrafish and pacu (*Piaractus mesopotamicus*), the results obtained with tambaqui may be compared to other Ostariophysi species.

## 2 MATERIAL AND METHODS

Tambaqui larvae were obtained by hormonally induced spawning from breeders kept in captivity, from the Balbina Fish Farming Station of the State Department of Rural Production (SEPROR), AM, Brazil (latitude −1.191; longitude −59.466). After fertilization, eggs were incubated in a 200 L cylinder‐conical incubator with constant water circulation (27.8 ± 0.2°C) until all larvae hatch. Then, larvae were transported to the Laboratory of Ecophysiology and Molecular Evolution of the Brazilian National Institute for Research of the Amazon (LEEM/INPA) in Manaus, AM, and the plastic bag containing all larvae was placed inside a 150 L holding tank filled with the same water used to fill the tanks inside each room. For approximately one hour, small amounts of this water was diluted inside the bag, for the initial acclimatization of larvae. After counting the exact number of larvae per tank (315 individuals), the batch was transferred to a 1 L recipient containing approximately 1/4 of water from the respective tank. Each recipient remained floating on each tank surface and, during one hour, small amounts of the tank‐water were slowly exchanged, before larvae were released into the tanks.

### 2.1 Experimental design

Larvae were exposed to the climate change scenarios predicted by the Fourth Special Report on Emission Scenarios (SRES) of the IPCC (IPCC, 2007), in four climate‐controlled rooms, each one with ten 9 L tanks (replicates) arranged in a water recirculation system. The conditions foreseen in the IPCC (2007) report were used in this study due to logistical issues required to update the controlled rooms to the environmental conditions foreseen in the IPCC 2014 report. Each room is equipped with an electronic high‐tech system with a set‐point for atmospheric temperature, humidity and CO_2_ concentration that updates every other minute. The control treatment simulated the current climatic conditions of a pristine tropical forest located in the main *campus* of INPA (from 10 October 2014 to 25 October 2014), given by sensors installed in the forest. The atmospheric conditions of the other three rooms simulating the climate change scenarios B1, A1B and A2 (IPCC, 2007) were real‐time adjusted considering the *current* conditions existing in the control room. Thus, based on the control treatment, the first scenario (*mild* or B1) had an increase of 1.8°C and 200 ppm of CO_2_ in its atmosphere; the second (*moderate* or A1B) had a 2.8°C and 400 ppm of CO_2_ increase; while the third and more severe scenario (*drastic* or A2) had a 3.4°C and 850 ppm increase in atmospheric CO_2_ concentration. The water in the recirculation system (which contained the experimental tanks) located inside each controlled room equilibrated with the local atmospheric conditions, after the period needed to reach the equilibrium. Therefore, variations of water temperature, CO_2,_ and pH throughout the experimental period occurred only due to the atmospheric variations inside the rooms. The water used to fill the recirculation system in each controlled room was previously collected in the Balbina Fish Farming Station and transported to the laboratory, thus avoiding sudden water quality exchange. Both the water temperature (°C) and dissolved O_2_ (mg/L) were registered daily, using an oximeter YSI (5512‐FT), while pH and CO_2_ concentration (ppm) in water were assessed every other day, using a pH meter UltraBASIC UB10 (Denver Instrument) and the colorimetric method of Boyd and Tucker (1992), respectively.

Tambaqui larvae were stocked in each 9 L tank at an initial density of 35 larvae/L and were fed *Artemia* nauplii in increasing quantities, as suggested by Jomori, Carneiro, Malheiros, and Portella (2003) for *P. mesopotamicus* larvae and adapted for tambaqui in our laboratory. Throughout the experiment, 10 larvae from each tank were sampled eleven times, at hatching up to the 16th day posthatching (dph); thus, in each sampling event, 100 larvae were collected from each treatment. All animals were anesthetized and euthanized in benzocaine solution (0.15 g/L), then fixed in a formalin solution at 4% for 48 hr and preserved in ethanol 70°GL.

Sampled larvae were weighted, measured and, at the end of the experiment (16th dph), the following zootechnical indexes were calculated: Specific Growth Rate (SGR) ((100 × {ln final weight − ln initial weight}/days of experiment)), Condition Factor (K) (weight/(length^*b*^), *b* = 3.57) and Mass Gain (MG) (final weight − initial weight). In addition, the yolk‐sac area of larvae with 0 to 6 dph was measured (mm^2^) using a stereoscope (Olympus SZX7) and an image analyzer software (CellSens Standard v.1.6).

After biometric evaluations, all larvae underwent a differential bone and cartilage staining process, following the methodology proposed by Potthoff (1984), excluding the bleaching step, because the larvae were too small and had no body pigmentation yet. After the staining process, larvae were preserved in glycerin and then analyzed for the skeletal development. Skeletal anomalies were registered by means of a digital photographic camera (Olympus DP26) attached to the stereoscope previously used to measure the animals, and were qualified (lordosis, scoliosis, kyphosis, malformation in vertebral bodies, upper jaw and lower jaw) and quantified as the presence (1) or absence (0) of skeleton anomalies.

This study was approved by the Ethics Committee on Animal Use of the Faculty of Agrarian and Veterinary Sciences of the São Paulo State University (CEUA/FCAV/UNESP), protocol nº 11714/14.

### 2.2 Statistical analysis

All data were tested as for error normality (Shapiro‐Wilk's test) and variance homoscedasticity (Levene's test) and are expressed as mean ± standard error (*SE*). An one‐way ANOVA, followed by a Dunnett's post hoc test (Dunnett, 1955) was performed to distinguish differences between the climate change and the current scenarios, regarding environmental variables. Zootechnical data were also evaluated by a one‐way ANOVA, followed by the Tukey's post hoc. For both tests, a 5% significance level was admitted and both were performed with the aid of the Software R (version 3.4.0, 2017).

Skeletal anomalies (categorical data) were evaluated by two different methods. At first, the incidence of anomalies was analyzed by *one‐way* ANOVA, followed by the Tukey's post hoc test. Thereafter, in order to seek existing associations between the climate change scenarios and the types of skeletal anomalies, an exploratory multivariate analysis of Simple Correspondence was applied, which was performed by means of the Chi‐square test. Besides the anomalies data, a Larval Quality Index (LQI) was calculated according to Boglione et al. (2009), considering the number of deformed larvae per treatment. At last, an exploratory Cluster analysis (by hierarchic method) was used to evaluate anomalies incidence. The Software STATISTICA 7.0 was used for all exploratory multivariate analysis.

## 3 RESULTS

**Figure 1:**
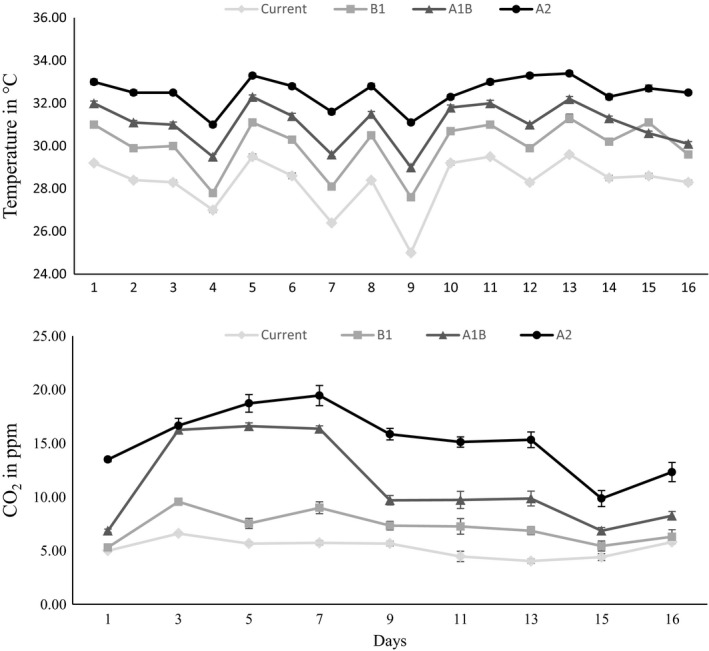
Water temperature and dissolved CO_2_ concentration in the controlled rooms throughout the experiment. Values are presented as mean (*n* = 10) ± *SE*, representing mean values obtained in the observation of 10 tanks per room

The climate‐controlled rooms efficiently simulated the different climate change scenarios predicted by the IPCC (IPCC, 2007) (Table 1). Water temperature and CO_2_ concentration inside the tanks are shown in Figure 1.

**Table 1:** Atmospheric temperature and CO_2_ concentration, and environmental variables of the water inside the climate‐controlled rooms used in the experiment, reflecting the future climatic conditions predicted by the IPCC. Values are presented as mean ± *SE*

Scenario	Atmospheric Temperature (°C)	Atmospheric CO_2_ (ppm)	Dissolved Oxygen (mg/L)	Water pH
Current	27.91 ± 2.07	498.9 ± 27.5	7.34 ± 0.09	7.10 ± 0.07
B1	29.96 ± 1.99*	704.2 ± 61.1*	7.07 ± 0.07	7.03 ± 0.09
A1B	30.87 ± 2.00*	900.4 ± 44.1*	6.88 ± 0.06*	6.96 ± 0.07
A2	32.95 ± 1.99*	1284.9 ± 50.1*	6.64 ± 0.06*	6.80 ± 0.09

Notes

Asterisks indicate significant differences (*p* < 0.05) between the *current* scenario and the predicted climate change scenarios, by the Dunnett's test.

Current: *current* environmental conditions; B1: *mild* scenario; A1B: *moderate* scenario; A2: *drastic* scenario.

The larvae exposed to *current* conditions and to the three climate change scenarios showed complete yolk‐sac absorption at 6 dph, which was statistically similar within treatments throughout time (*p* > 0.05). At the end of the experiment, larvae exposed to *current* conditions and to the A2 scenario were smaller (*p* < 0.0001) in weight and length, compared to B1 and A1B scenarios, a result that was also verified in the specific growth rate (Supporting Information Figure S1). Despite the similar growth performance of larvae exposed to *current* and the A2 scenarios, survival rates were higher in the *current* scenario and lower within the climate change scenarios, especially for the A2 scenario (the most severe one), which is the most severe one (Supporting Information Figure S1). It is remarkable that the condition factor was significantly higher in larvae reared both in the *current* conditions (2.64 ± 0.09, *p* < 0.001) and A1B (2.41 ± 0.14, *p* < 0.001) scenario, while the larvae exposed to the A2 presented the lowest values (1.88 ± 0.12, *p* < 0.001) (Supporting Information Figure S1).

**Figure 2:**
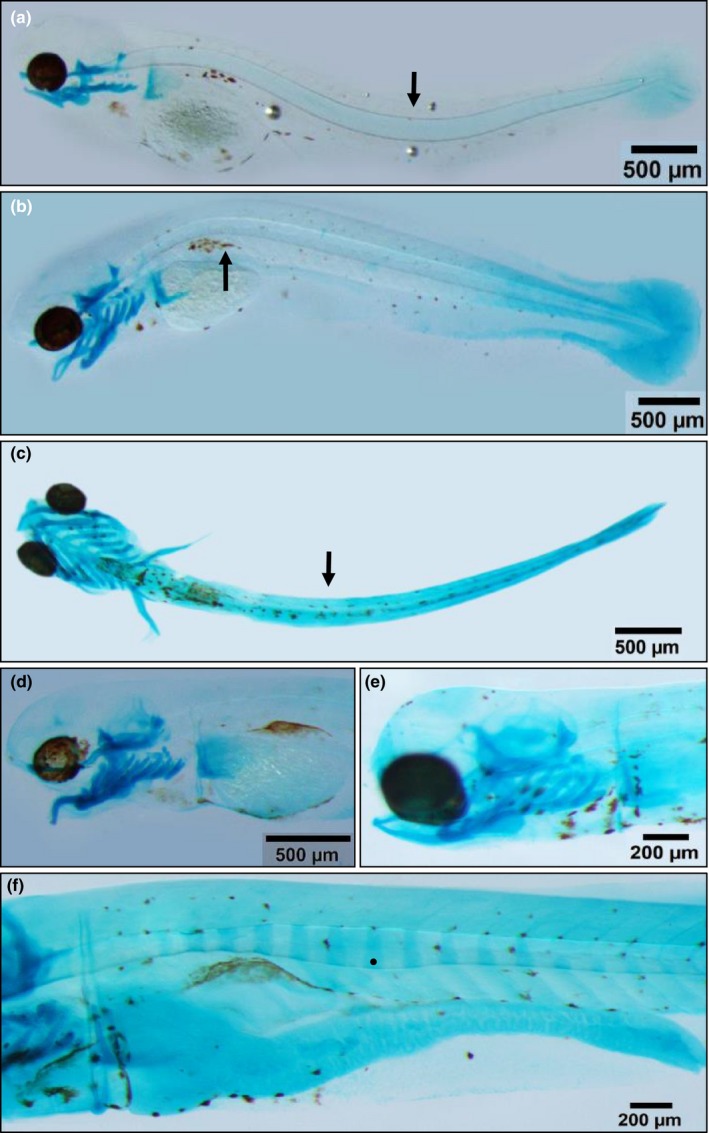
Skeletal anomalies registered in tambaqui larvae exposed to different climate change scenarios. (a) Lordosis, characterized by a dorso‐ventral curvature of the notochord (5 dph); (b) kyphosis, characterized by a ventral‐dorsal curvature of the notochord (5 dph); (c) scoliosis, characterized by a latero‐lateral curvature of the notochord (5 dph); (d) upper‐jaw malformation (9 dph); (e) lower‐jaw malformation (9 dph); (f) malformation of the future vertebral bodies, evidenced by an anterior flattening of the cartilage model (16 dph)

Bone mineralization was not observed in larvae exposed to both *current* conditions and to the studied climate change scenarios. However, the incidence of skeletal anomalies increased from the control to the drastic scenario (Table 2), resulting in a highest occurrence of anomalies in larvae exposed to A2 scenario (39.91%), in comparison with all other treatments. The type of anomaly varied among scenarios, with lordosis, considered as the most severe one (Figure 2a), often registered for larvae exposed to A2 scenario.

**Table 2:** Incidence (%) of skeletal anomalies found in tambaqui larvae exposed to different climate change scenarios

Skeletal anomalies	Current	B1	A1B	A2
Lordosis (%)	4.59^c^	4.77^c^	7.43^bc^	15.19^a^
Scoliosis (%)	0.88^b^	3.01^a^	3.71^a^	3.71^a^
Kyphosis (%)	0.70^c^	1.93^bc^	3.01^b^	6.36^a^
Upper Jaw (%)	3.01^c^	7.24^a^	5.65^b^	7.06^a^
Lower Jaw (%)	5.30^a^	3.35^b^	6.55^a^	6.55^a^
Ʃ of deformed larvae (%)	15.38^c^	21.28^bc^	34.82^b^	39.91^a^

Letters indicate significant differences (*p* < 0.05) regarding skeletal anomalies in the different climatic scenarios, according to the Tukey's test.

Current: *current* environmental conditions; B1: *mild* scenario; A1B: *moderate* scenario; A2: *drastic* scenario.

Still concerning the malformations in the notochord, kyphosis was the second most frequent anomaly in larvae of the treatment A2 (Figure 2b), while scoliosis was the third most current anomaly in these larvae (Figure 2c). Jaw anomalies have also been found in tambaqui larvae (Figure 2d,e) exposed to the climate change scenarios predicted for the end of the century (IPCC, 2007). Other anomalies were also observed, such as malformations of the vertebrae cartilage model (Figure 2f), but as it appeared in a very small frequency, these were not considered for statistical analysis.

**Figure 3:**
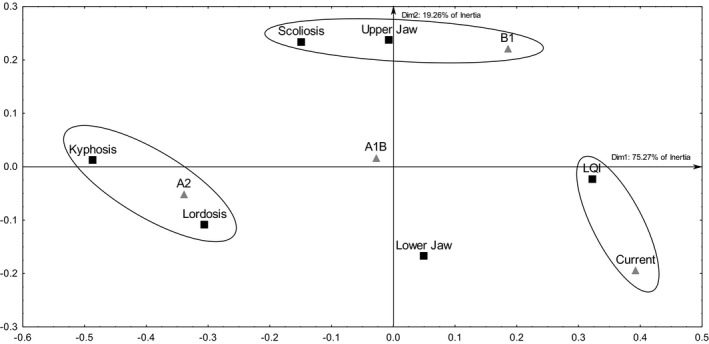
Perceptual map showing associations between climate change scenarios (triangles) and skeletal anomalies (squares), revealed by the correspondence multivariate analysis. Black ellipses indicate statistically significant associations between a climatic condition and skeletal anomalies (*p* < 0.05). Current: *current* environmental conditions; B1: *mild* scenario; A1B: *moderate* scenario; A2: *drastic* scenario; LQI: larval quality index

Based on the incidence of skeletal anomalies, the cluster analysis revealed an expected proximity between the A1B and A2 scenarios, both being different from the *current* and B1 conditions (Supporting Information Figure S2). In addition, the simple correspondence analysis revealed possible associations between the predicted climate change scenarios and types of skeletal anomalies (Table 3), whereas a better larval quality index was observed for the larvae exposed to the *current* conditions (Figure 3).

**Table 3:** Observed minus expected frequencies obtained after data standardization by the Chi‐square test

Scenarios	Lordosis	Scoliosis	Kyphosis	Upper Jaw	Lower Jaw	LQI
Current	−9.3010	−7.4821	−9.2622	−8.3542	**6.0109**	28.3886*
B1	−14.0376	**2.4895**	−4.4174	11.5255*	−8.8874	**13.3274**
A1B	−3.3718	**4.9569**	−0.0457	−0.5874	**6.1672**	−7.1191
A2	26.7103*	**0.0357**	13.7254*	−2.5837	−3.2907	−34.5969

The asterisks indicate the statistically significant associations, which presented normality deviations above 1.96, indicating *p* values above 0.05, by the Tukey's test.

Positive values (bold) indicate possible associations between climate change scenarios and skeletal anomaly.

Current: *current* environmental conditions; B1: *mild* scenario; A1B: *moderate* scenario; A2: *drastic* scenario; LQI: larval quality index.

## 4 DISCUSSION

Skeletal anomalies appear both in natural and artificial environments, with higher frequencies occurring in abnormal climatic conditions, as demonstrated in this study. Tambaqui larvae will not develop its skeleton in a normal way when facing climate change expected for the end of the century, especially under moderate and extreme conditions of high temperatures, dissolved CO_2_ and water acidity, as observed in the A1B and A2 scenarios (IPCC, 2007). These climatic conditions resulted in severely structurally compromised skeletons, with marked notochord curvatures (lordosis, scoliosis and kyphosis), certainly affecting the animal's welfare and its *fitness* both in natural and artificial environments (Boglione et al., 2009, 2013; Izquierdo et al., 2010; Lopes, Freitas, Jomori, Carneiro, & Portella, 2014). Poorly developed structures in the craniofacial skeleton were also observed (e.g., crooked upper and lower jaws), which may hinder larvae growth by directly affecting food capture (Boglione et al., 2009), as verified in the A2 scenario.

The aforementioned anomalies were also found in larvae deriving from wild broodstocks, as verified by Lopes et al. (2014) in pacu *Piaractus mesopotamicus*, a phylogenetically closely related species of tambaqui. In this case, the appearance of anomalies is unpredictable, as several factors are involved in fish ontogeny. On the contrary, in artificial environments, such as the climate‐controlled rooms used in this study, the differential occurrence of skeletal anomalies were given mainly by different conditions inside each room. It is noteworthy that almost 40% of the larvae exposed to the A2 scenario showed at least one type of bone malformation, while in the remaining controlled rooms, the occurrence of anomalies was significantly lower. In pacu larvae obtained from wild broodstock and reared in captivity, the incidence of cranial and column anomalies were 10.9% and 15.8%, respectively (Lopes et al., 2014).

The main causes of skeletal anomalies in fish relate to genetic (Georgakopoulou et al., 2010), nutritional (Cahu, Zambonino‐Infante, & Takeuchi, 2003) and environmental factors (Boglione et al., 2013) or handling methods (Izquierdo et al., 2010; Wargelius, Fjelldal, & Hansen, 2005). Thus, we argue that the differential occurrence of anomalies verified in all treatments was given by environmental factors, seen that all larvae were obtained from the same pair of breeders, feed was supplied as the routine protocol for tambaqui larviculture and was identical within all animals, and the adopted handling methods were also identical among the treatments. Thus, genetic, nutritional or handling factors were not the causes that triggered the appearance of such anomalies. The differences among the controlled rooms were exclusively their atmospheric temperatures and CO_2_ concentrations, which reflected in differential combination of water temperature and acidity inside the tanks.

The ontogeny of organs and systems of fish larvae may be affected by inappropriate abiotic factors (e.g., temperature), especially when an organism has genetic predisposition to develop malformations (Kause et al., 2007). In this context, it is possible that both increased water temperature, dissolved CO_2,_ and acidity may have influenced the animals’ growth and skeletal development. As observed by Pimentel et al. (2014), the first life stages of fish—when larvae do not have yet developed an sufficient ion‐regulatory system, represent a critical period for the organisms’ ecological success. When exposed to climate change scenarios with increased CO_2_ and temperature, the lack of a good regulation capacity may lead to metabolic depression, growth reduction, a compromised skeletal ontogeny and, consequently, differential survival rates (Ou et al., 2015; Pimentel et al., 2014; Pittman et al., 2013; Pörtner, Langenbuch, & Reipschlager, 2004; Rosa & Seibel, 2008; Seibel & Walsh, 2001). This may be the reason for the increasing occurrences of anomalies in fish exposed to the climate change scenarios (B1, A1B, and A2) in the climate‐controlled rooms. In a similar manner, survival rates were also differential among scenarios, being lower in the A2 scenario (24.7 ± 1.0%), comparing to the others. Altricial larvae, such as the tambaqui larvae used in this study, do not possess a completely developed physiological and metabolic apparatus at their early stages of development. In this sense, high mortalities rates may be observed when these organisms face climate change, because early hatched larvae might not be yet adapted to such climatic variations, as suggested by Pittman et al. (2013).

Different incidences of skeletal anomalies were verified in the four predicted climate change scenarios. These results are in accordance to the zootechnical parameters evaluated, showing a lower performance of larvae exposed to the most extreme scenario (A2, 6.84 ± 0.9 mm length and 1.79 ± 0.1 mg) in comparison with others. Lopes et al. (2014) showed that bone mineralization starts at 6 dph in *P. mesopotamicus* (5.5 mm total length), first through the dentary bone, then by the ninth vertebrae followed by the final portion of the notochord, so it was expected that tambaqui larvae would undergo the same process, as they are closely related species. Although, in marine species, several authors (Boglione et al., 2009, 2013; Cahu et al., 2003; Noble et al., 2012; Wargelius et al., 2005) indicate that under different rearing conditions (e.g., larvae density, feeding, light regime and water quality parameters), this event may be delayed. Another hypothesis about the delay or the nonmineralization of the tambaqui bone structures concerns to the water used in the experiment, which came from Balbina reservoir. This water is typically acid and deficient in calcium carbonate, an essential element in the composition and formation of mineralized bone structures (Feely et al., 2004). The pH values registered throughout the experiment were close to neutrality, with slight variations among treatments, however, such variations are meaningful, considering that slight reduction in water pH may be very problematic for fish species, especially during their early stages (Caldeira & Wickett, 2003; Pimentel et al., 2014). Therefore, new studies must be performed in order to investigate the time for mineralization of the tambaqui's bone structures in controlled and natural environments, as the water pH and mineral composition influences this process.

The proximity revealed by the cluster analysis between the A1B and A2 scenarios reinforces the hypothesis that the species will be severely affected by moderate or drastic climatic changes, as also reported by Oliveira and Val (2017). When analyzing the significant associations found between severe skeletal anomalies and climate change scenarios, especially the most drastic one (A2), and between larval quality index and the larvae exposed to current conditions, we assume that the predicted climate changes, regardless of its dimension will affect the life cycle of the species *C. macropomum*. The tambaqui is a strict freshwater species and the appearance of skeletal anomalies facing climate change were similar to what was found in other studies with marine species (Dionísio et al., 2012; Georgakopoulou et al., 2010; Lall & Lewis‐McCrea, 2007; Pimentel et al., 2014), demonstrating that future climatic variations will similarly affect both freshwater and marine species.

Throughout its reproduction season, the tambaqui migrates to muddy, nutrients‐rich waters with a typical stable temperature (around 28°C). After spawning, newly hatched larvae grow in floodplains where the variations of temperature and dissolved oxygen are wider (Gomes, Simões, & Araújo‐Lima, 2013). Thus, these organisms must be able to tolerate a certain range of environmental conditions, without compromising its homeostasis. The thermal tolerance range of a species varies depending on animal's age (Wilson & Nagler, 2006), and this range was not yet studied for tambaqui. Our results suggested that over the first days of development, tambaqui larvae grows in a healthier way and with higher survival rates in temperatures close to 28°C, despite presenting greater growth (weight and length) in higher temperatures, as also verified in the marine species *Theragra chalcogramma* (Hurst, Fernandez, & Mathis, 2013).

Anthropogenic activities are currently one of the greatest problems in the Amazon basin, causing habitat fragmentation and river courses deviations as consequences of deforestation, dam constructions, excessive exploitation of natural stocks and pollution (Castello et al., 2013). These activities directly influence fish populations, as well as other aquatic species, and certainly the predicted climate variations for the end of the century will augment such impacts, by increased or decreased precipitation patterns or by water quality variations, as previously quoted (IPCC, 2007, 2014), even leading to extinction of vulnerable species (Hugueny, Movellan, & Belliard, 2011). However, it is possible that some species are better prepared to face mild or moderate environmental changes, as verified by Luo, Guan, Li, and Jin (2013) with two eel species (*Anguilla marmorata* and *A. bicolor*), which developed well in slightly elevated temperatures, but not in drastic conditions. Tambaqui juvenile were considered as “partially” adapted to mild and moderate predicted climate change (B1 and A1B scenarios), seen that they showed physiological adaptations in blood parameters, such as increased cortisol and glucose concentrations, which enabled their survival in these scenarios (Oliveira & Val, 2017). Indeed, larvae grew more under these intermediate conditions than when exposed to the current and drastic conditions (*current* and A2 scenarios), but conversely developed a greater number of skeletal anomalies and possibly were not in a higher welfare status than the *current* condition larvae, reflected by the condition factor. The influences that climate change will exert in this species and its response to such impacts involves complex mechanisms (Prado‐Lima & Val, 2016) that must be studied in order to better understand how this important species will respond to future climatic variations. A recent study demonstrated that one of the main current challenges for Amazonian species is the mitigation of anthropogenic influences in this biome (Foster, Falter, McCulloch, & Clode, 2016). In this sense, urgent conservation actions should be taken into account in order to reduce such impacts on this biome (Assunção, Gandour, & Rocha, 2015). In conclusion, the environmental alterations caused by predicted climate change for the end of the century will affect growth, survival and the skeletal development of *Colossoma macropomum* larvae, an important Amazonian food species.

## CONFLICT OF INTEREST

The authors declare no conflict of interest.

## AUTHORS CONTRIBUTION

IGL participated in data collection, analysis, and writing the manuscript. TBAD and JTK participated in data collection analysis. MCP and ALV participated in data analysis and writing the manuscript.

## DATA ACCESSIBILITY

The authors declare that the supplementary data (growth performance results and the Dendrogram deriving from the cluster multivariate analysis) of the present study will be placed in Dryad repository, as well as the environmental data collected inside the climate‐controlled rooms (both atmospheric and water temperature and CO_2_ concentration), and pictures of deformed and normal larvae. The DOI provided by the Dryad Repository is https://doi.org/10.5061/dryad.m2bj3nc.

## References

[ece34429-bib-0001] Assunção, J. , Gandour, C. , & Rocha, R. (2015). Deforestation slowdown in the Brazilian Amazon: Prices or policies? Environmental and Development Economics, 20, 697–722. 10.1017/S1355770X15000078

[ece34429-bib-0002] Bignami, S. , Sponaugle, S. , & Cowen, R. K. (2013). Response to ocean acidification in larvae of a large tropical marine fish, *Rachycentron canadum* . Global Change Biology, 19(4), 996–1006. 10.1111/gcb.12133 23504878

[ece34429-bib-0003] Bird, C. B. , & Mabee, P. M. (2003). Developmental morphology of the axial skeleton of the zebrafish, *Danio rerio* (Ostariophysi: Cyprinidae). Developmental Dynamics, 228(3), 337–357. 10.1002/dvdy.10387 14579374

[ece34429-bib-0004] Boglione, C. , Gisbert, E. , Gavaia, P. , Witten, P. E. , Moren, M. , Fontagné, S. , & Koumoundouros, G. (2013). Skeletal anomalies in reared European fish larvae and juveniles. Part 2: Main typologies, occurrences and causative factors. Reviews in Aquaculture, 5(s1), S121–S167. 10.1111/raq.12016

[ece34429-bib-0005] Boglione, C. , Marino, G. , Giganti, M. , Longobardi, A. , Marzi, P. D. , & Cataudella, S. (2009). Skeletal anomalies in dusky grouper *Epinephelus marginatus* (Lowe 1834) juveniles reared with different methodologies and larval densities. Aquaculture, 291(1–2), 48–60. 10.1016/j.aquaculture.2009.02.041

[ece34429-bib-0006] Boyd, C. E. , & Tucker, C. S. (1992). Water quality and pond soil analyses for aquaculture (p. 183). Auburn, AL: Auburn University.

[ece34429-bib-0007] Byrne, M. (2011). Impact of ocean warming and ocean acidification on marine invertebrate life history stages: Vulnerabilities and potential for persistence in a changing ocean. Oceanography and Marine Biology: An Annual Review, 49, 1–42. 10.1201/b11009-2

[ece34429-bib-0008] Byrne, M. , Soars, N. , Selvakumaraswamy, P. , Dworjany, S. A. , & Davis, A. R. (2010). Sea urchin fertilization in a warm, acidified and high pCO_2_ ocean across a range of sperm densities. Marine Environmental Research, 69(4), 234–239. 10.1016/j.marenvres.2009.10.014 19913293

[ece34429-bib-0009] Cahu, C. , Zambonino‐Infante, J. , & Takeuchi, T. (2003). Nutritional components affecting skeletal development in fish larvae. Aquaculture, 227(1–4), 245–258. 10.1016/S0044-8486(03)00507-6

[ece34429-bib-0010] Caldeira, K. , & Wickett, M. E. (2003). Anthropogenic carbon and ocean pH. Nature, 425, 365 10.1038/425365a 14508477

[ece34429-bib-0011] Castello, L. , McGrath, D. G. , Hess, L. L. , Coe, M. T. , Lefebvre, P. A. , Petry, P. , … Arantes, C. C. (2013). The vulnerability of Amazon freshwater ecosystems. Conservation Letters, 6(4), 217–229. 10.1111/conl.12008

[ece34429-bib-0012] Dionísio, G. , Campos, C. , Valente, L. M. P. , Conceição, L. E. C. , Cancela, M. L. , & Gavaia, P. J. (2012). Effect of egg incubation temperature on the occurrence of skeletal deformities in *Solea senegalensis* . Journal of Applied Ichthyology, 28, 471–476. 10.1111/j.1439-0426.2012.01996.x

[ece34429-bib-0013] Dunnett, C. W. (1955). A multiple comparison procedura for comparing several treatments with a control. Journal of the American Statistical Association, 50, 1096–1121. 10.1080/01621459.1955.10501294

[ece34429-bib-0014] Estêvão, M. D. , Silva, N. , Redruello, B. , Costa, R. , Gregório, S. , Canário, A. V. M. , & Power, D. M. (2011). Cellular morphology and markers of cartilage and bone in the marine teleost *Sparus aurata* . Cell and Tissue Research, 343(3), 619–635. 10.1007/s00441-010-1109-y 21234603

[ece34429-bib-0015] Feely, R. A. , Sabine, C. L. , Lee, K. , Berelson, W. , Kleypas, K. , Fabry, V. J. , & Millero, F. J. (2004). Impact of anthropogenic CO_2_ on the CaCO_3_ system in the oceans. Science, 305(5682), 362–366. 10.1126/science.1097329 15256664

[ece34429-bib-0016] Foster, T. , Falter, J. L. , McCulloch, M. T. , & Clode, P. L. (2016). Ocean acidification causes structural deformities in juvenile coral skeletons. Science Advances, 2(2), 1–7. 10.1126/sciadv.1501130 PMC4788479 26989776

[ece34429-bib-0017] Georgakopoulou, E. , Katharios, P. , Divanach, P. , & Koumoundouros, G. (2010). Effect of temperature on the development of skeletal deformities in Gilthead seabream (*Sparus aurata* Linnaeus, 1758). Aquaculture, 308(1–2), 13–19. 10.1016/j.aquaculture.2010.08.006

[ece34429-bib-0018] Gomes, L. C. , Simões, L. N. , & Araújo‐Lima, C. A. R. M. (2013). Tambaqui (*Colossoma macropomum*) In Baldisserotto B. & Gomes L. C. (Eds.), Espécies Nativas para Piscicultura no Brasil (pp. 175–204). Santa Maria, Brazil: UFSM.

[ece34429-bib-0019] Hamre, K. , Yúfera, M. , RØnnestad, I. , Boglione, C. , Conceição, L. E. C. , & Izquierdo, M. (2013). Fish larval nutrition and feed formulation: Knowledge gaps and bottlenecks for advances in larval rearing. Reviews in Aquaculture, 5(s1), S26–S58. 10.1111/j.1753-5131.2012.01086.x

[ece34429-bib-0020] Hoffmann, A. A. , & Sgrò, C. M. (2011). Climate change and evolutionary adaptation. Nature, 470, 479–485. 10.1038/nature09670 21350480

[ece34429-bib-0021] Hugueny, B. , Movellan, A. , & Belliard, J. (2011). Habitat fragmentation and extinction rates within freshwater fish communities: A faunal relaxation approach. Global Ecology and Biogeography, 20(3), 449–463. 10.1111/j.1466-8238.2010.00614.x

[ece34429-bib-0022] Hurst, T. P. , Fernandez, E. R. , & Mathis, J. T. (2013). Effects of ocean acidification on hatch size and larval growth of walleye Pollock (*Theragra chalcogramma*). ICES Journal of Marine Science, 70(4), 812–822. 10.1093/icesjms/fst053

[ece34429-bib-0023] IPCC (2007). Climate Change 2007: The physical science basis In Core Writing Team, Pachauri R. K., & Reisinger A. (Eds.), Contribution of Working Groups I, II and II to the Fourth Assessment Report of the Intergovernmental Panel on Climate Change (104 pp.). Geneva, Switzerland: IPCC.

[ece34429-bib-0024] IPCC (2014). Climate Change 2014: Synthesis Report In Core Writing Team, Pachauri R. K. & Meyer L.A. (Eds.), Contribution of Working Groups I, II and II to the Fifth Assessment Report of the Intergovernmental Panel on Climate Change (151 pp). Geneva, Switzerland: IPCC.

[ece34429-bib-0025] Ishimatsu, A. , Kikkawa, T. , Hayashi, M. , Lee, K. S. , & Kita, J. (2004). Effects of CO_2_ on marine fish: Larvae and adults. Journal of Oceanography, 60(4), 731–741. 10.1007/s10872-004-5765-y

[ece34429-bib-0026] Izquierdo, M. S. , Socorro, J. , & Roo, J. (2010). Studies on the appearance of skeletal anomalies in red porgy: Effect of culture intensiveness, feeding habits and nutritional quality of live preys. Journal of Applied Ichthyology, 26(2), 320–326. 10.1111/j.1439-0426.2010.0149.x

[ece34429-bib-0027] Johnston, I. A. , & Hall, T. E. (2004). Mechanisms of muscle development and responses to temperature change in fish larvae. American Fisheries Society Symposium, 40, 85–116.

[ece34429-bib-0028] Jomori, R. K. , Carneiro, D. J. , Malheiros, E. B. , & Portella, M. C. (2003). Growth and survival of pacu *Piaractus mesopotamicus* (Holmberg, 1887) juveniles reared in ponds of at different initial larviculture periods indoors. Aquaculture, 221(1–4), 277–287. 10.1016/S0044-8486(03)00069-3

[ece34429-bib-0029] Kause, A. , Ritola, O. , & Paananen, T. (2007). Changes in the expression of genetic characteristics across cohorts in skeletal deformations of farmed salmonids. Genetics Selection Evolution, 39(5), 529–543. 10.1051/gse:2007019 PMC2682804 17897595

[ece34429-bib-0030] Kochhann, D. , Campos, D. F. , & Val, A. L. (2015). Experimentally increased temperature and hypoxia affect stability of social hierarchy and metabolism of the Amazonian cichlid *Apistogramma agassizii* . Comparative Biochemistry and Physiology Part A: Molecular & Integrative Physiology, 190, 54–60. 10.1016/j.cbpa.2015.09.006 26387464

[ece34429-bib-0031] Lall, S. P. , & Lewis‐McCrea, L. M. (2007). Role of nutrients in skeletal metabolism and pathology in fish – An overview. Aquaculture, 267(1–4), 3–19. 10.1016/j.aquaculture.2007.02.053

[ece34429-bib-0032] Lopes, T. S. , Freitas, T. M. , Jomori, R. K. , Carneiro, D. J. , & Portella, M. C. (2014). Skeletal anomalies of pacu, *Piaractus mesopotamicus*, larvae from a wild‐caught broodstock. Journal of the World Aquaculture Society, 45(1), 15–27. 10.1111/jwas.12092

[ece34429-bib-0033] Luo, M. , Guan, R. , Li, Z. , & Jin, H. (2013). The effects of water Temperature on the survival, feeding, and growth of the juveniles of *Anguilla marmorata* and *A. bicolor pacifica* . Aquaculture, 400–401, 61–64. 10.1016/j.aquaculture.2013.03.003

[ece34429-bib-0034] Malhi, Y. , Roberts, J. T. , Betts, R. A. , Killeen, T. J. , Li, W. , & Nobre, C. A. (2008). Climate change, deforestation and the fate of the Amazon. Science, 319(5860), 169–172. 10.1126/science.1146961 18048654

[ece34429-bib-0035] Miller, G. M. , Kroon, F. J. , Metcalfe, S. , & Munday, P. L. (2015). Temperature is the evil twin: Effects of increased temperature and ocean acidification on reproduction in a reef fish. Ecological Applications, 25(3), 603–620. 10.1594/PANGAEA.836664 26214908

[ece34429-bib-0036] Noble, C. , Jones, H. A. C. , Damsgård, B. , Flood, M. J. , Midling, K. Ø. , Roque, A. , … Cottee, S. Y. (2012). Injuries and deformities in fish: Their potential impacts upon aquacultural production and welfare. Fish Physiology and Biochemistry, 38(1), 61–83. 10.1007/s10695-011-9557-1 21918861

[ece34429-bib-0037] Oliveira, A. M. , & Val, A. L. (2017). Effects of Climate Scenarios on the growth and physiology of the Amazonian Fish tambaqui (*Colossoma macropomum*) (Characiformes: Serrasalmidae). Hydrobiologia, 789(1), 167–178. 10.1007/s10750-016-2926-0

[ece34429-bib-0038] Ou, M. , Hamilton, T. J. , Eom, J. , Lyall, E. M. , Gallup, J. , Jiang, A. , … Brauner, C. J. (2015). Responses of pink salmon to CO_2_‐induced aquatic acidification. Nature Climate Change, 5, 950–957. 10.1038/NCLIMATE2694

[ece34429-bib-0039] Parmesan, C. (2006). Ecological and evolutionary responses to recent climate change. Annual Review of Ecology, Evolution and Systematics, 37, 637–669. 10.1146/annurev.ecolsys.37.091305.110100

[ece34429-bib-0040] Pimentel, M. S. , Faleiro, F. , Dionísio, G. , Repolho, T. , Pousão‐Ferreira, P. , Machado, J. , & Rosa, R. (2014). Defective skeletogenesis and oversized otoliths in fish early stages in a changing ocean. The Journal of Experimental Biology, 217, 2062–2070. 10.1242/jeb.092635 24625652

[ece34429-bib-0041] Pittman, K. , Yúfera, M. , Pavlidis, M. , Geffen, A. J. , Koven, W. , Ribeiro, L. , … Tandler, A. (2013). Fantastically plastic: Fish larvae equipped for a new world. Reviews in Aquaculture, 5(s1), S224–S267. 10.1111/raq.12034

[ece34429-bib-0042] Portella, M. C. , Leitão, N. J. , Takata, R. , & Lopes, T. S. (2012). Alimentação e Nutrição de Larvas In Fracalossi D. M., & Cyrino J. E. P. (Eds.), Nutriaqua: Nutrição e alimentação de espécies de interesse para a aquicultura brasileira (pp. 185–216). Florianópolis, Brazil: Ministério da Pesca e Aquicultura.

[ece34429-bib-0043] Pörtner, H. O. , Langenbuch, M. , & Reipschlager, A. (2004). Biological impact of elevated ocean CO_2_ concentrations: Lessons from animal physiology and Earth history. Journal of Oceanography, 60(4), 705–718. 10.1007/s10872-004-5763-0

[ece34429-bib-0044] Potthoff, T. (1984). Clearing and staining techniques In Moser H. G., Richards W. J., Cohen D. M., Fahay M. P., Kendall J. R., & Richardson S. L. (Eds.), Ontogeny and systematics of fishes. American Society of Ichthyologists and Herpetologists. Special Publication (pp. 35–37).

[ece34429-bib-0045] Prado‐Lima, M. , & Val, A. L. (2016). Transcriptomic characterization of Tambaqui (*Colossoma macropomum*, Cuvier, 1818) exposed to three climate change scenarios. PLoS ONE, 11(3), 1–21. 10.1371/journal.pone.0152366 PMC4809510 27018790

[ece34429-bib-0046] Rosa, R. , Pimentel, M. S. , Boavida‐Portugal, J. , Teixeira, T. , Trubenbach, K. , & Diniz, M. (2012). Ocean warming enhances malformations, premature hatching, metabolic suppression and oxidative stress in the early life stages of a keystone squid. PLoS ONE, 7(6), e38282 10.1371/journal.pone.0038282 22701620 PMC3368925

[ece34429-bib-0047] Rosa, R. , & Seibel, B. A. (2008). Synergistic effects of Climate‐related variables suggest future physiological impairment in a top oceanic predator. Proceedings of the National Academy of Sciences of the United States of America, 105(52), 20776–20780. 10.1073/pnas.0806886105 19075232 PMC2634909

[ece34429-bib-0048] Rosa, R. , Trubenbach, K. , Pimentel, M. S. , Boavida‐Portugal, J. , Faleiro, F. , Baptista, M. , … Repolho, T. (2014). Differential impacts of ocean acidification and warming on winter and summer progeny of a coastal squid (*Loligo vulgaris*). The Journal of Experimental Biology, 217, 518–525. 10.1242/jeb.096081 24523499

[ece34429-bib-0049] Rosa, R. , Trubenbach, K. , Repolho, T. , Pimentel, M. , Faleiro, F. , Boavida‐Portugal, J. , … Pörtner, H. O. (2013). Lower hypoxia thresholds of cuttlefish early life stages living in a warm acidified ocean. Proceedings of the Royal Society B: Biological Sciences, 280(1768), 1–7. 10.1098/rspb.2013.1695 PMC3757987 23926158

[ece34429-bib-0050] Rosenzweig, C. , Karoly, D. , Vicarelli, M. , Neofotis, P. , Wu, Q. , Casassa, G. , … Imeson, A. (2008). Attributing physical and biological impacts to anthropogenic climate change. Nature, 453, 353–358. 10.1038/nature06937 18480817

[ece34429-bib-0051] Seibel, B. A. , & Walsh, P. J. (2001). Potential impacts of CO_2_ injection on deep‐sea biota. Science, 294, 319–320. 10.1126/science.1065301 11598290

[ece34429-bib-0052] Tseng, Y. C. , Hu, M. Y. , Stumpp, M. , Lin, L. T. , Melzner, F. , & Hwang, P. P. (2013). CO_2_‐driven seawater acidification differentially affects development and molecular plasticity along life history of fish (*Oryzias latipes*). Comparative Biochemistry and Physiology, Part A, 165(2), 119–130. 10.1016/J.CBPA.2013.02.005 23416137

[ece34429-bib-0053] Wargelius, A. , Fjelldal, P. G. , & Hansen, T. (2005). Heat shock during early somitogenesis induces caudal vertebral column defects in Atlantic salmon (*Salmo salar*). Development Genes and Evolution, 215(7), 350–357. 10.1007/s00427-005-0482-0 15798920

[ece34429-bib-0054] Wilson, S. M. , & Nagler, J. J. (2006). Age, but not salinity, affects the upper lethal temperature limits for juvenile walleye (*Sander vitreus*). Aquaculture, 257(1–4), 187–193. 10.1016/j.aquaculture.2005.10.045

