# Supplementary material for: Predicted 2100 climate scenarios affects growth and skeletal development of tambaqui (Colossoma macropomum) larvae
Source: Ecol Evol. 2018 Oct 3;8(20):10039–48. doi: 10.1002/ece3.4429 (PMC6206194; doi:10.1002/ece3.4429)
Supplement: Supplementary file 1 [file ECE3-8-10039-s001.docx]

**S1.** Mean values of total length (mm), humid mass (mg), specific growth rate (%.day^-1^), condition factor, yolk-sac area (mm^2^) and survival (%) throughout the experiment (16 dph).

| Variables | Current | B1 | A1B | A2 |
| --- | --- | --- | --- | --- |
| Total Length |  |  |  |  |
| 0 dph | 2.862 ± 0.011 | 2.862 ± 0.011 | 2.862 ± 0.011 | 2.862 ± 0.011 |
| 1 dph | 3.742 ± 0.033 ^b^ | 3.694 ± 0.041 ^b^ | 3.665 ± 0.026 ^b^ | 3.911 ± 0.033 ^a^ |
| 2 dph | 4.530 ± 0.033 | 4.606 ± 0.024 | 4.566 ± 0.028 | 4.543 ± 0.015 |
| 3 dph | 4.998 ± 0.023 ^a^ | 4.865 ± 0.096 ^ab^ | 4.887 ± 0.016 ^b^ | 4.970 ± 0.019 ^a^ |
| 4 dph | 5.087 ± 0.021 | 5.121 ± 0.082 | 5.103 ± 0.016 | 5.131 ± 0.014 |
| 5 dph | 5.256 ± 0.014 | 5.188 ± 0.023 | 5.194 ± 0.018 | 5.228 ± 0.017 |
| 7 dph | 5.373 ± 0.012 ^a^ | 5.280 ± 0.015 ^b^ | 5.214 ± 0.016 ^c^ | 5.288 ± 0.028 ^bc^ |
| 10 dph | 5.444 ± 0.014 ^a^ | 5.331 ± 0.021 ^b^ | 5.326 ± 0.019 ^b^ | 5.372 ± 0.025 ^ab^ |
| 13 dph | 6.502 ± 0.082 ^a^ | 6.370 ± 0.061 ^ab^ | 6.460 ± 0.0112 ^a^ | 6.127 ± 0.055 ^b^ |
| 16 dph | 6.494 ± 0.072 ^b^ | 7.567 ± 0.068 ^a^ | 7.804 ± 0.0111 ^a^ | 6.846 ± 0.090 ^b^ |
| Humid Mass |  |  |  |  |
| 0 dph | 0.486 ± 0.006 | 0.486 ± 0.006 | 0.486 ± 0.006 | 0.486 ± 0.006 |
| 1 dph | 0.569 ± 0.007 | 0.547 ± 0.009 | 0.535 ± 0.010 | 0.541 ± 0.013 |
| 2 dph | 0.544 ± 0.004 | 0.541 ± 0.004 | 0.544 ± 0.010 | 0.517 ± 0.015 |
| 3 dph | 0.589 ± 0.005 ^a^ | 0.535 ± 0.006 ^b^ | 0.538 ± 0.003 ^b^ | 0.582 ± 0.004 ^a^ |
| 4 dph | 0.631 ± 0.007 | 0.622 ± 0.004 | 0.620 ± 0.006 | 0.624 ± 0.005 |
| 5 dph | 0.683 ± 0.008 ^a^ | 0.641 ± 0.004 ^b^ | 0.619 ± 0.0053 ^c^ | 0.610 ± 0.008 ^c^ |
| 7 dph | 0.698 ± 0.006 ^ab^ | 0.639 ± 0.008 ^c^ | 0.664 ± 0.0059^bc^ | 0.712 ± 0.007 ^a^ |
| 10 dph | 0.837 ± 0.011 | 0.773 ± 0.018 | 0.785 ± 0.0011 | 0.831 ± 0.042 |
| 13 dph | 1.225 ± 0.075 | 1.173 ± 0.047 | 1.278 ± 0.101 | 1.155 ± 0.103 |
| 16 dph | 1.821 ± 0.034 ^c^ | 3.168 ± 0.091 ^b^ | 3.698 ± 0.128 ^a^ | 1.795 ± 0.129 ^c^ |
| Yolk-sac Area |  |  |  |  |
| 0 dph | 0.709 ± 0.007 | 0.709 ± 0.007 | 0.709 ± 0.007 | 0.709 ± 0.007 |
| 3 dph | 0.480 ± 0.010 | 0.499 ± 0.008 | 0.457 ± 0.009 | 0.482 ± 0.009 |
| 6 dph | 0.144 ± 0.007 | 0.157 ± 0.009 | 0.136 ± 0;016 | 0.097 ± 0.005 |
| Specific Growth Rate | 8.76 ± 0.80 ^c^ | 14.37 ±1.20 ^b^ | 16.17 ± 1.57 ^a^ | 9.52 ± 3.33 ^c^ |
| Condition Factor (K) | 2.64 ± 0.09 ^a^ | 2.14 ± 0.05^b^ | 2.41 ± 0.14 ^ab^ | 1.88 ± 0.12^c^ |
| Survival | 29.3 ± 0.3 ^a^ | 27.5 ± 0.6 ^ab^ | 26.3 ± 0.7 ^b^ | 24.7 ± 1.0 ^b^ |

Means followed by distinct letters indicate statistical differences among treatments (p < 0.05) according to the Tukey’s test. **Current**: *current* environmental conditions; **B1**: *mild* scenario; **A1B**: *moderate* scenario; **A2**: *drastic* scenario.

**S2.** Dendrogram obtained by the hierarchic method and Euclidian distances, revealing a proximity relation between the A1B and A2 scenarios, while an expected distance from these to to B1 and *current* climatic conditions.
